# Properties and Uses of Papain

**Published:** 1897-12

**Authors:** 


A MEMOIR on the properties and uses of Papain is included in a volume just published on "Indigestion" by Dr. G. Herschell. He describes the origin and nature of the ferment, and cites the evidence on which its powerful peptonizing influence was established. Experiments conducted with a view to deciding whether the substance produced true peptone or not, resulted in conclusive proof that the former was the case. For practical purposes, says Dr. Hershell, as a digestive ferment to be given medicinally, papain presents the following advantages over pepsin and pancreatin:

1. It will convert or digest many more times its own weight of meat than they are able to.

2. It can be used when pepsin and pancreatin are contraindicated or powerless.

This latter, as known, is the case when the stomach contents are too concentrated, or insufficiently acid. Under these conditions pepsin is of little or no value, while papain acts energetically. Other advantages of papain are expressed as follows;

3. As regards albuminoids it combines in itself the joint action of pepsin and pancreatin.

4. It can be given combined with acids, alkalies or antiseptics, as indicated by the demands of the case.

5. It has a local action on the stomach that pepsin has not.

6. It is not so repulsive to the mind as pepsin, as it is purely vegetable.

All these properties being taken into consideration, papain is regarded as being indicated in deficiency of the gastric juice, excess of unhealthy mucus in the stomach, irritable condition of that viscus, and duodenal dyspepsia.

The effect of papain is thus two-fold, local on the stomach itself, or physiologic, removing unhealthy mucus, stimulating the secretion of gastric juice and relieving pain; and chemical, in peptonizing the food, and assisting the natural ferments in the work of digestion.

